# Treatment Delay and Type of Retraction Affect the Surgical Treatment of Distal Biceps Tendon Ruptures: A Quantitative Analysis of 123 Patients

**DOI:** 10.3390/healthcare13161977

**Published:** 2025-08-12

**Authors:** Giuseppe Giannicola, Sebastien Prigent, Alessandra Colozza, Davide Blonna, Andrea Amura, Pasquale Sessa

**Affiliations:** 1 Department of Anatomic, Histological, Forensic Medicine and Orthopedic Sciences, Sapienza University of Rome, Piazzale A. Moro 5-7, 00162 Rome, Italy; giuseppe.giannicola@uniroma1.it (G.G.); andrea.amura95@gmail.com (A.A.); p.sessa@hotmail.it (P.S.); 2 Department of Orthopedics and Traumatology—Ospedale degli Infermi, Viale Stradone 9, 48018 Faenza, Italy; alex.colozza@gmail.com; 3 Department of Orthopedics and Traumatology—Ospedale Umberto I, Via Magellano 1, 00128 Turin, Italy; davide.blonna@virgilio.it

**Keywords:** distal biceps tendon rupture, distal biceps tendon repair, distal biceps tendon reconstruction, tendon graft, distal biceps tendon retraction, surgical timing

## Abstract

**Introduction**: This study aimed to quantitatively evaluate the effect of the trauma to surgery time interval (Tt-Ts) and the type of tendon retraction (unretracted vs. coiled) in distal biceps tendon ruptures (DBTRs) on the choice between primary tendon repair (PR) and tendon reconstruction with graft (RG). **Materials and Methods**: In total, 123 patients with surgically treated DBTRs were analyzed. Patients were divided into three groups: Group I—acute (75 patients with Tt-Ts < 21 days), Group II—subacute (20 patients with Tt-Ts between 21 and 45 days), and Group III—chronic (28 patients with Tt-Ts > 45 days). The type of surgical treatment (PR vs. RG) was evaluated in each group. The type of tendon retraction (unretracted vs. coiled) was analyzed in subacute and chronic lesions. A statistical analysis was performed. **Results:** The mean Tt-Ts interval in the overall sample was 59.3 days; in Group I, it was 9 days (range, 2–20); in Group II, it was 29 days (range, 22–42); and in Group III, it was 196 days (range, 45–1095). PR was performed in 100%, 90%, and 29% of the patients in Groups I, II, and III, respectively. Coiled tendons were found in 60% and 71% of patients in Groups II and III, respectively. Among patients with coiled tendons, 2 and 20 received RG in Groups II and III, respectively. The Tt-Ts and the type of retraction were significantly associated with the choice of surgical treatment (PR vs. RG), with statistical differences between Group III and the other two groups (*p* < 0.05). A cut-off of 43.5 days following injury was found to predict the need to perform RG with an accuracy, sensitivity, and specificity of 94%, 100%, and 92%, respectively. The likelihood of receiving RG rather than PR increased each day by 6%. **Conclusions**: Treatment delay significantly affects the choice of surgical technique in DBTRs. PR is feasible in 98% of acute and subacute ruptures, whereas RG is necessary in 70% of chronic ruptures. The type of tendon retraction affects the choice of treatment only in chronic lesions, as coiled tendons always require RG.

## 1. Introduction

Distal biceps brachii tendon ruptures (DBTRs) mainly affect male patients between the fourth and sixth decade, with an incidence of 1.2–2.5 per 100,000 persons/year [1]. DBTRs require surgery in active patients, as conservative treatment is associated with chronic pain and functional impairment, particularly in supination strength [2,3]. The best timing for surgical treatment is undoubtedly the acute phase, before excessive scarring and degeneration have occurred and when primary reinsertion (PR) of the tendon into the radial tuberosity is less technically demanding. However, late presentation of patients or requests for additional imaging examinations often delay surgery, which results in DBTRs being treated in the chronic setting. As PR in the chronic setting may not be feasible in some cases owing to tendon retraction and degeneration, a reconstruction with a graft (RG) is required [4,5,6,7,8,9,10,11,12,13,14,15,16,17,18,19]. No guidelines are as yet available for chronic DBTRs, and few studies have attempted to identify preoperative parameters that can guide surgeons in selecting the most appropriate technique; the trauma to surgery time interval (Tt-Ts), the degree of tendon retraction, the degeneration of the tendon stump, the presence of an intact lacertus fibrosus, and a peritendinous sheath are all factors believed to significantly affect the feasibility of PR [3,11,20,21,22,23]. However, the choice between PR and RG is currently still based above all on the surgeon’s personal experience, as few quantitative data exist [8,23,24].

The hypothesis of this study was that Tt-Ts and the type of tendon retraction are significantly associated with the choice of surgical technique in DBTRs. In particular, we hypothesized that coiled tendon type retraction and delay in surgical treatment are strong predictors of the need for tendon reconstruction rather than primary repair. We designed a multicenter study to quantitatively assess how Tt-Ts and the type of retraction affect the choice between PR and RG. A secondary aim was to perform a narrative literature review of this topic to determine whether a therapeutic algorithm could be drawn up.

## 2. Materials and Methods

A retrospective multicenter study was performed by including patients from three elbow surgery centres. A series of patients with acute, subacute, and chronic DBTRs treated surgically between 2014 and 2020 was assessed. Three skilled elbow surgeons (GG, AC, and DB) performed all of the procedures.

The exclusion criteria were (1) follow-up < 12 months, (2) associated local injuries, (3) sharp penetrating injuries, (4) connective tissue disorders, (5) long-term history of corticosteroid intake or chemotherapy, (6) patients with previous failed DBTR surgery, and (7) partial DBTR.

In total, 131 patients were identified (129 males and 2 females). However, 8 patients were lost to follow-up, leaving 123 eligible patients. The patients were divided into three groups according to the Tt-Ts: Group I (75 acute injuries with Tt-Ts < 21 days), Group II (20 subacute injuries with Tt-Ts between 21 and 45 days), and Group III (28 chronic injuries with Tt-Ts > 45 days). Although controversies still exist in the literature on what time interval constitutes an acute, subacute, or chronic injury [1,25], we defined a Tt-Ts of 21 days as acute [6,24] and a Tt-Ts > 45 days as chronic [26]; consequently, DBTRs were considered subacute when the Tt-Ts was between 22 and 45 days.

**Figure 1:**
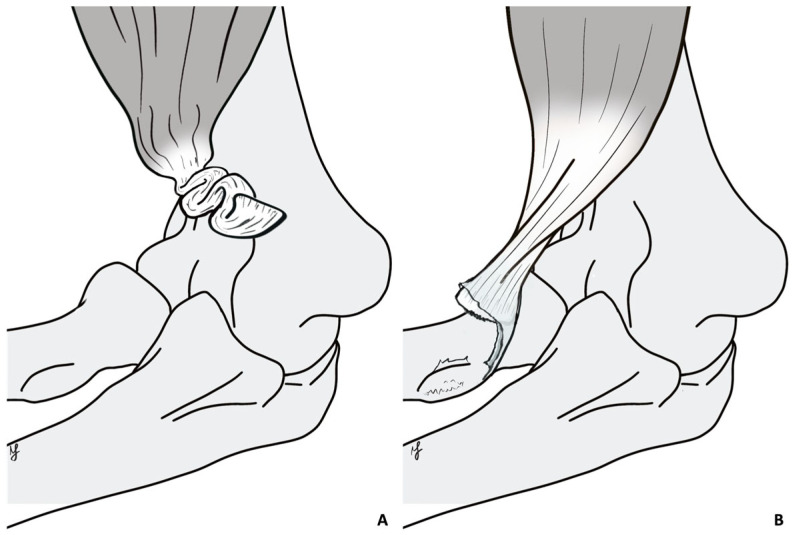
The figure shows the type of tendon retraction. (**A**) Coiled tendon retraction pattern: the tendon is severely retracted and coiled on itself due to a fibrous scar; the tendon is significantly shortened. (**B**) Linear tendon retraction pattern: the tendon is still stretched due to an intact lacertus fibrosus and/or tendon sheath, conserving its length. Original drawing.

The patients’ general characteristics and clinical data were collected. The type of retraction of the tendon stump (unretracted vs. coiled, Figure 1) was evaluated intraoperatively. Functional outcomes were assessed using the Mayo Elbow Performance Score (MEPS), the Disabilities of the Arm, Shoulder and Hand (DASH) questionnaire, and a visual analogue scale (VAS) for pain. Elbow range of motion (ROM) was measured in extension (E), flexion (F), pronation (P), and supination (S) using a goniometer. Muscle strength in flexion and supination was graded using the Medical Research Council (MRC) scale, ranging from 0 (no movement) to 5 (normal strength). Complications were noted. Written informed consent, in accordance with the Declaration of Helsinki, was obtained from all the participants. Ethics committee approval was also obtained (n° 0953/2023, Rif. 7382).

### 2.1. Surgical Technique Note

The type of surgical procedure (PR vs. RG) was decided intraoperatively following musculotendinous unit release and mobilization of the tendon stump. A PR was always performed when the tendon could be drawn close to the radial tuberosity, even in extreme flexion [27]. A Krackow-type suture with a #2 non-absorbable wire (Fiber-wire, Arthrex^®^) was performed, followed by tenodesis with transosseous sutures or 5 mm metallic suture anchors (Corkscrew, Arthrex©). In particular, of the 101 patients who received PR, 63 were treated with a double-incision technique and a transosseous suture [22], while the remaining 38 were treated with a single-incision technique and tenodesis with two anchors [28,29]. Of the 22 patients who received RG, 14 were treated with a double-incision technique and a transosseous suture, while the remaining 8 were treated with a single-incision technique and 2 anchors. The types of graft used were 13 autologous semitendinosus, 5 autologous lacertus fibrosus, 1 homologous posterior tibial, 2 homologous gracilis, and 1 homologous peroneal brevis.

### 2.2. Statistical Analysis

A descriptive analysis of all of the variables assessed was performed. Fischer’s F test was used to assess differences between groups for the type of surgical procedure received and the type of tendon retraction. The ANOVA with post hoc Tukey’s test was used to assess differences between groups for the variables considered. Crude and adjusted logistic regression models were used to calculate the odds ratio for receiving RG versus PR according to the Tt-Ts interval (Equations (1) and (2)). On the basis of the point estimate of the odds ratio between the crude and the adjusted logistic regression models, we computed the absolute value of delta variation of the odds ratio (OR), as shown in Equation (3). We considered the crude logistic regression model rather than the adjusted logistic regression when delta variation dropped to below 1%. These equations model the probability that a patient will require graft reconstruction (RG) rather than primary repair (PR) based on the time between trauma and surgery. Logistic regression was used to estimate the strength of this association, and the resulting model allowed us to determine a time-based threshold (cut-off) for guiding the surgical decision. To choose between the crude and adjusted model, we calculated the delta variation in odds ratios (Equation (3)); when the variation was less than 1%, the crude model was considered sufficient for further analysis.

**Figure 2:**
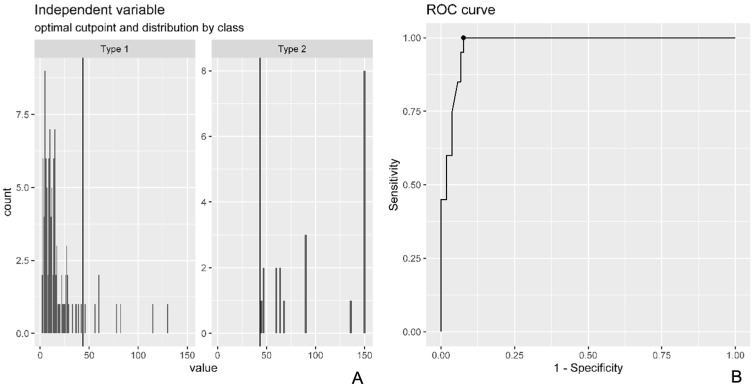
(**A**) The figure shows the raw (type 1) and adjusted (type 2) logistic regression models used to calculate the odds ratio (OR) to receive RG or PR. (**B**) The figure shows the ROC curve and relative metrics used to determine the optimal cut-off for the model used in the statistical analysis.

**Figure 3:**
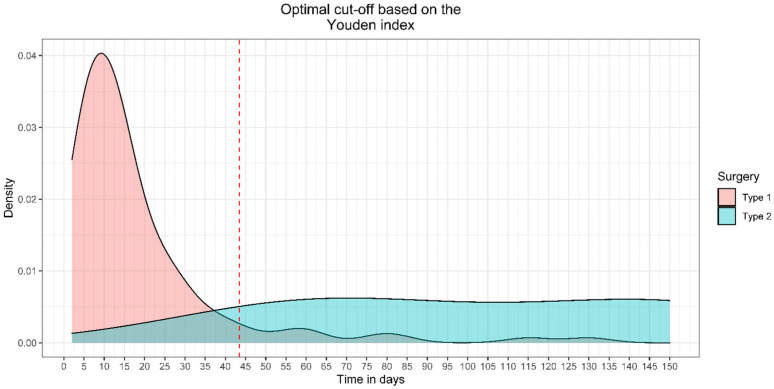
The Youden Index Point was used to calculate the optimal cut-off. At 43.5 days, there was an accuracy of 0.936, a sensibility of 1, and a specificity of 0.924 to correctly classify patients who underwent PR rather than RG. Surgery type 1 = PR (Primary Repair); surgery type 2 = RG (Reconstruction with Graft).

We computed a Receiver Operating Characteristic (ROC) in order to determine the optimal cut-off point at which surgeons decided to use RG rather than PR using the crude or adjusted logistic regression model based on the results of Equation (3) (Figure 2A,B). For optimal cut-off point identification, we used the Youden Index Point (Figure 3). The ROC curve was plotted along with a density plot of the time to surgery stratified by surgery type. The ROC curve was used to identify the trauma to surgery time point that best distinguishes patients who are likely to need graft reconstruction from those suitable for primary repair. The Youden Index was applied to find the cut-off with the highest combination of sensitivity and specificity, supporting clinical decision making with a quantitative threshold.

(1) $$\left(crude\;logistic\;regression\right):\;Ln\left(\frac{Prg}{1-Prg}\right)=B0+B1\;time$$

(2) $$\left(adjusted\;logistic\;regression\right):Ln\;\left(\frac{Prg}{1-Prg}\right)=B0+B1time+B2sex+B3age$$

(3) $$Delta\;variations=\frac{Odds\;ratio\;adjusted-Odds\;ratio\;crude}{Odds\;ratio\;crude}$$

The significance level was set at alpha = 0.05. SPSS 20 (IBM, Chicago, IL, USA) for Windows was used for the statistical analysis.

### 2.3. Literature Review

A qualitative literature review of the past 20 years (January 2015 to January 2025) was performed using the MEDLINE database (PubMed) with the following keyword combinations: “distal biceps tendon repair OR reconstruction; chronic distal biceps tendon; distal biceps tendon primary repair; distal biceps tendon reconstruction with graft.” The following inclusion criteria were used: (1) studies on acute, subacute, and chronic DBTRs treated surgically with PR and/or RG; (2) literature reviews; and (3) imaging studies that correlate the pathological features of chronic tendon injury with the surgical technique used and/or the Tt-Ts. The following exclusion criteria were applied: (1) lack of data on the surgical technique used; (2) unreported Tt-Ts; (3) case reports; (4) papers not published in English or published in non-indexed journals.

## 3. Results

The general characteristics and clinical data are shown in Table 1 and Table 2. No significant differences were found for age, side, or gender between the three groups examined (*p* > 0.05).

PR was performed in all of the patients with an acute and subacute tendon injury, except in two cases in Group II (10%) in whom the tendon was coiled after a Tt-Ts of 36 days in one patient and 42 days in the other. In the other 18 subacute cases treated with PR, the tendon stump was found unretracted in 8 cases, while it was coiled in 10 cases. The lacertus fibrosus was ruptured in 12 out of 20 patients in Group II (10 coiled and 2 unretracted). In 20 of the 28 patients (71.4%) in Group III, RG was performed after a mean Tt-Ts of 243 days (range: 45–1095). In these 20 patients, the myotendinous unit was markedly shortened and the tendon coiled and severely degenerated; only a hypertrophic myotendinous junction was found in these 20 cases. The lacertus fibrosus in 5 out of these 20 patients appeared intact and thickened and was used as a graft. A PR was performed in the remaining 8 of the 28 patients in Group III after a mean Tt-Ts of 78 days (range: 46–130); the biceps tendon was always found unretracted, close to the radial tuberosity and adherent to the peritendinous sheath, which appeared thickened and still inserted in the radial tuberosity.

A significant between-group difference (*p* = 0.001) emerged in the surgical technique used, with a higher number of RG being performed in Group III (71%) than in Groups II (10%) and I (0%). The type of tendon retraction was significantly associated with the choice of surgical technique in Groups II and III, with a higher number of RG being performed in coiled tendons and when the lacertus fibrosus was ruptured (*p* = 0.00001).

When we analyzed the correlation between the Tt-Ts and the type of surgery, the crude model revealed that the odds ratio (OR) for RG versus PR was 1.059 (95% CI: 1.038–1.090) for each day added to the Tt-Ts. In the adjusted model, the OR was 1.062 (95%CI: 1.040–1.096). Consequently, the delta change was 0.38%, which was used as a basis for the crude model to identify the optimal temporal cut-off for the choice of surgical procedure to perform in DBTRs; this cut-off was 43.5 days. This means that 43.5 days after injury, there is an accuracy of 0.936, sensitivity of 1, and specificity of 0.924 in correctly identifying subjects who should undergo RG rather than PR (Figure 3). The multivariate logistic regression model yielded a 6% increase per day in the likelihood of receiving RG rather than PR.

The clinical results are shown in detail in Table 3.

The complications observed in this study cohort are shown in detail in Table 4.

### Literature Review Results

The review yielded 71 eligible articles, and 46 of the 71 papers were excluded following application of the exclusion criteria. RG was performed after a mean Tt-Ts of 182.9 days (range: 33.8–644.4), whereas PR was performed after a mean Tt-Ts of 26.3 days (range: 7.3–97.2). The results of the review are shown in detail in Table 5.

## 4. Discussion

The main aim of this study was to quantitatively evaluate the effect of delayed treatment and type of tendon retraction on the choice of surgical technique in DBTRs. A large series of surgically treated patients with acute, subacute, and chronic DBTR was analyzed. The results showed that the Tt-Ts is significantly associated with the choice of treatment, with PR being possible in the majority of acute and subacute patients while RG was required in more than 70% of chronic patients. The type of tendon retraction was the main factor affecting the choice of surgical technique in the chronic group; RG was always required in chronic patients with “coiled” tendons, whereas PR was always still feasible in those with “unretracted” tendons.

Several biomechanical and clinical studies have highlighted the benefits of surgical treatment in acute DBTRs, with a significant recovery of muscle strength and resistance. These encouraging results have resulted in conservative treatment being reserved for elderly, low-demand patients or cases with severe comorbidities [7,9,11,27,29].

Despite these findings, a significant number of symptomatic active patients with chronic DBTRs who have not undergone surgical treatment in the acute phase are still being observed. The muscle belly retraction and tendon degeneration resulting from delayed surgery often prevent PR in the chronic setting, which leads to the need for more complex reconstructive techniques requiring the use of grafts [17,21,25,37]. A lack of guidelines for chronic DBTRs that may preoperatively guide the surgeon represents a major issue. Indeed, the choice between PR and RG is based on the individual surgeon’s experience, with the decision often being made intraoperatively, as was the case in the present study.

The absence of guidelines for chronic DBTRs is mainly due to the lack of quantitative data on the prognostic pathoanatomic factors that may affect the choice of surgical procedure, which include muscle belly trophism, tendon quality and dimensions before injury, rupture level, type of rupture (partial or complete), associated lacertus fibrosus tear, degree of degeneration, and proximal retraction of the tendon, as well as muscle belly atrophy after trauma [21,22,24,27]. Most of these factors are unquestionably related to the time interval between rupture and surgery; indeed, the time factor is mentioned in several studies and widely believed to be a significant prognostic factor.

The results of this study quantitatively demonstrate that delayed treatment significantly affects the choice of surgical technique, which is a widely shared opinion. In particular, significant differences emerged between acute/subacute lesions and chronic lesions. PR was adopted for 98% of the injuries treated within 43 days of trauma. This finding shows that PR can be used within this time interval to treat DBTRs without the risk of encountering significant technical difficulties, which questions the widely held belief that surgery becomes more complex and the results less predictable if not performed within 4 weeks [20]. Only two patients received RG within 43 days of injury owing to a marked shortening of the muscular belly associated with a retracted, coiled, and degenerated tendon. In contrast, all of the chronic injuries that required RG revealed a severely coiled, shortened, and degenerated tendon both at the MRI and intraoperatively. However, it is noteworthy that eight patients in Group III, which included cases with a Tt-Ts of up to 130 days, received a PR. These eight patients displayed an intact lacertus fibrosus and a tendon stump that was adherent to the tendon sheath near the radial tuberosity. In our cohort, preoperative MRI findings correlated well with intraoperative observations. Coiled tendon morphology on MRI consistently matched severely retracted and degenerated stumps requiring graft reconstruction, whereas unretracted tendons observed on MRI were typically suitable for primary repair. This supports the role of MRI as a valuable preoperative planning tool in chronic DBTRs.

When the muscular contraction during trauma causes the rupture of the lacertus fibrosus and the peritendinous sheath, marked proximal retraction of the tendon stump may occur; in these cases, the tendon often rolls up on itself, forming a coiled “ball of wool” that is surrounded, in the subsequent weeks, by inflammatory tissue that rapidly causes its degeneration. In the first 6 weeks, the initial scar tissue can, in the majority of cases, be removed through careful dissection, thereby “uncoiling” the tendon stump and recovering its original length, which allows PR to be performed. As time goes by, the “ball of wool” degenerates, thereby making it impossible to “uncoil.” This pathological progression explains the observation in chronic cases of retracted tendons characterized by a thickened myotendinous junction alone, which invariably required graft reconstruction. In contrast, chronic cases in which the tendon remained close to the radial tuberosity and adherent to the peritendinous sheath, which were often associated with an intact lacertus fibrosus, displayed a lesser degree of tendon degeneration, thereby allowing PR to be performed.

These findings are consistent with the study published in 2019 by Luokkala et al. [23] in which the authors described the use of the Hook test as a predictive tool to preoperatively determine the need for a graft. In that study, the Hook test demonstrated a sensitivity ranging from 80% to 86% for detecting distal biceps tears. The sensitivity was lower in cases where the lacertus fibrosus was found to be intact (45%) during the operation and in partial tears (30%). The results highlighted how the result of the Hook test, in conjunction with the time delay between the initial injury and the operation, could be used to plan reconstruction for the patient. The need for a graft rose from 20% to 75% when there was a delay of over eight weeks and the Hook test was positive (i.e., coiled tendon). In contrast, when the Hook test was negative (i.e., unretracted tendon with intact lacertus fibrosus and/or peritendinous sheath), the likelihood of receiving a graft reconstruction was low (20%), even after 12 weeks. The results of our study quantitatively support these findings and reflect the clinical experiences of numerous other authors, suggesting that Tt-Ts is a useful clinical parameter in preoperative planning.

In this study, the regression analysis showed that 43 days after trauma, the accuracy, sensitivity, and specificity in preoperatively establishing the need for patients to undergo RG are, respectively, 94%, 100%, and 92%; moreover, the likelihood of receiving RG increases by 6% every day. In the first months of the chronic phase, the ROC curve displayed a “grey area” in which the time factor alone was not sufficient to preoperatively establish which technique is most likely required. Based on the intraoperative observations of our subacute and chronic sample, we believe that this “grey area” may be considerably reduced by analyzing the type of tendon retraction. In particular, when the tendons are “coiled,” RG is always required later than 45 days after trauma, whereas PR can still be performed when the tendon remains unretracted following rupture; these findings can be gleaned from the Hook test and a preoperative MRI [23,24,38]. The type of tendinous retraction, a factor that is often not adequately described, may also explain the apparent conflicting results in the literature. In particular, our literature review revealed that the mean Tt-Ts for PR and RG was, respectively, 26.3 days (range: 7.3–97.2) and 182.9 days (range: 33.8–644.4), thereby confirming that PR can be performed in the majority of acute and subacute cases. Although the distinction between acute and subacute cases may appear to be of limited value, some major technical difficulties that do not occur in the acute phase may be encountered in the subacute phase, a case in point being the need to address coiled tendons.

This study has two main limitations: firstly, the unavoidable variability inherent in a multicenter study, and, secondly, the relatively small sample size, particularly that of the chronic cases (n = 28). However, it should be borne in mind that when diseases are rare, multicenter studies can provide larger samples from which meaningful conclusions can be drawn. Moreover, it is worth considering that the current series of subacute and chronic patients represents one of the largest in the literature to date. A third apparent limitation of the study may be the fact that different surgical approaches and techniques were used; however, it must be stressed that the primary aim of this study was to assess the effect of delayed treatment on the choice between PR and RG and not the viability of the different surgical techniques. Our clinical results nevertheless demonstrate that excellent clinical results were achieved in acute, subacute, and chronic patients regardless of the time elapsed and the surgical technique used. The 43.5-day cut-off identified in this study requires prospective clinical validation in larger cohorts to confirm its applicability in routine clinical practice.

## 5. Conclusions

Treatment delay significantly affects the choice of treatment in DBTRs. PR is feasible in 98% of cases within 43 days, whereas RG is necessary in 70% of chronic ruptures. In particular, a cut-off of 43 days after injury was associated with high accuracy, sensitivity, and specificity when adopted to predict the need for RG. The pattern of tendon retraction influences treatment choice only in the chronic phase, where coiled tendons consistently require graft reconstruction (RG). In contrast, unretracted tendons may still be suitable for primary repair (PR), even several years after the trauma. The combined assessment of Tt-Ts and the tendon retraction pattern on MRI is crucial for enabling the surgeon to appropriately plan surgical treatment in chronic DBTRs.

## Figures and Tables

**Table 1:** Demographics, trauma to surgery interval, and surgical technique stratified by group.

Variable (Range)	Whole Sample (n = 123)	Group I (n = 75)	Group II (n = 20)	Group III (n = 28)	*p*=
Age (range)	46 (29–64)	46.4 (31–63)	44.4 (34–62)	46.4 (29–64)	0.59
Sex					0.53
M	121	74	20	27	
F	2	1	0	1	
Tt-Ts—days (range)	53.9 (2–1095)	9.5 (2–20)	29.4 (22–42)	196 (45–1095)	0.001 I vs. II *p* = 0.7 I vs. III *p* = 0.001 II vs. III *p* = 0.001
PR (n)	101	75	18	8	0.001
RG (n)	22	0	2	20	

Tt-Ts: Trauma to Surgery interval; PR: Primary Repair; RG: Reconstruction with Graft.

**Table 2:** Patients’ characteristics and time to surgery interval stratified according to the surgical technique.

Groups			
Variables	PR (n = 101)	RG (n = 22)	*p*=
Age (range)	45.71 (31–62)	48 (29–64)	0.24
Sex	100 M; 1 F	21 M; 1 F	0.5
Side	R 58; L 43	R 13; L 9	0.88
Arm dominance	58	11	0.52
Tt-Ts (range)	18.16	224.5 (36–1095)	0.001

R: right; L: left; Tt-Ts: Trauma to Surgery interval; PR: Primary Repair; RG: Reconstruction with Graft.

**Table 3:** Muscle strength, ROM, and functional scores in the whole sample and stratified according to the three groups and between PR and RG.

Variable	Whole Sample (n = 123)	Group I (n = 75)	Group II (n = 20)	Group III (n = 28)	*p*=	PR (n = 101)	RG (n = 22)	*p*=
Strength in flexion (range)	4.9 (4–5)	5 (5–5)	5 (5–5)	4,9 (4–5)	0.005 I vs. II *p* = 0.1 I vs. III *p* = 0.004 II vs. III *p* = 0.04	4,98 (4–5)	4,95 (4–5)	0.48
Strength in supination (range)	4.9 (4–5)	5 (5–5)	4,9 (4–5)	4.86 (4–5)	0.001 I vs. II *p* = 0.17 I vs. III *p* = 0.001 II vs. III *p* = 0.45	4.97 (4–5)	4.8 (4–5)	0.005
ROM E° (range)	0° (0–10°)	0° (0–10°)	1° (0–10°)	1° (0–10°)	0.002 I vs. II *p* = 0.005 I vs. III *p* = 0.03 II vs. III *p* = 0.7	0.35° (0–10°)	1.136° (0–10°)	0.08
ROM F° (range)	136° (120–160°)	137° (120–160°)	136° (120–150°)	135° (120–145°)	0.5	136.7° (120–160°)	135.9° (130–140°)	0.71
ROM P° (range)	83° (30–90°)	85° (30–90°)	81° (70–90°)	82° (50–90°)	0.09	83.7° (60–90)	83.18° (70–90°)	0.8
ROM S° (range)	83° (0–90°)	85° (0–90°)	79° (60–90°)	82° (60–90°)	0.054	83.44° (60–90°)	83.4° (70–90°)	0.98
MEPS (range)	97 (70–100)	98 (85–100)	96 (85–100)	95 (70–100)	0.03 I vs. II *p* = 0.4 I vs. III *p* = 0.02 II vs. III *p* = 0.62	97.33 (70–100)	96.59 (85–100)	0.58
DASH (range)	1.25 (0–50.8)	1.0 (0–50.8)	0.3 (0–7.5)	2.15 (0–10)	0.92	1.13 (0–50.8)	1.33 (0–6.7)	0.92

ROM: range of motion; E: extension; F: flexion; P: pronation; S: supination; MEPS: Mayo Elbow Performance Score; DASH: disability of the arm and shoulder; PR: Primary Repair; RG: Reconstruction with Graft.

**Table 4:** Complications observed in the study cohort.

(**A**) Complications by treatment timing group				
**Group**	**HO**	**LACN Paresthesia**	**Superficial Wound Infections**	**Total Complications**
Acute	3	4	0	7
Subacute	0	2	2	4
Chronic	0	3	0	3
Total	3	9	2	14
(**B**) Complications by surgical technique				
**Surgical Technique**	**HO**	**LACN Paresthesia**	**Superficial Wound Infections**	**Total Complications**
Primary repair (PR)	3	8	2	13
Reconstruction with graft (RG)	0	1	0	1
Total	3	9	2	14

HO = Heterotopic Ossifications; LACN = lateral antebrachial cutaneous nerve.

**Table 5:** Literature review.

Article	Patients N°	PR vs. RG	Tt-Ts PR Mean (Range)	Tt-Ts RG Mean (Range)
Anakawaze et al., 2018 [30]	18	PR	33 (9–75)	-
Bosman et al., 2012 [31]	6	PR	78 (35–116)	-
Butler et al., 2023 [32]	45	PR	20 (6–84)	-
Caputo et al., 2016 [21]	12	RG	-	106 (14–617)
Cross et al., 2013 [4]	7	RG	-	175 (84–392)
Darlis et al., 2006 [5]	7	RG	-	196 (84–315)
Dillon et al., 2011 [6]	27	PR	53 (5–182)	-
Ford et al., 2018 [33]	970 (932 + 38)	PR and RG	21 (7–35)	59 (21–238)
Frank et al., 2019 [7]	35 (16 + 19)	PR and RG	37 (25–49)	273 (30–571)
Giacalone et al., 2015 [8]	23	PR	6 (1–19)	-
Goyal et al., 2020 [9]	11	RG	-	152 (30–395)
Hallam et al., 2004 [10]	9	RG	-	124 (76–197)
Haverstock et al., 2017 [11]	48	PR	23 (4–49)	-
Hendy et al., 2020 [12]	138 (92 + 46)	PR and RG	35 (3–446)	116 (15–1095)
Jain et al., 2024 [34]	66	PR	33 (7–130)	-
Khan et al., 2008 [35]	17	PR	19 (2–120)	-
McKee et al., 2005 [36]	53	PR	16 (2–84)	-
Morrel et al., 2012 [13]	12	RG	-	185 (42–476)
Morrey et al., 2014 [27]	46	PR	53 (1–784)	-
Phadnis et al., 2016 [14]	21	RG	-	760 (30–2920)
Samra et al., 2020 [15]	24	PR	45 (4–284)	-
Schmidt et al., 2024 [16]	30	PR	71 (42–204)	-
Snir et al., 2013 [17]	90	RG	-	140 (37–244)
Wiley et al., 2006 [18]	7	RG	-	119 (35–296)
Zeman et al., 2020 [19]	20	PR	70 (28–294)	-

## Data Availability

The data presented in this study are available upon request from the corresponding author due to privacy.
